# Struggling with Fitting in: Clients Mixed Experiences of Receiving Job Support and Getting a Job When Participating in Individual Placement and Support in Norway

**DOI:** 10.1007/s10926-024-10206-x

**Published:** 2024-05-23

**Authors:** Liv Grethe Kinn, Larry Davidson, Ketil Joachim Oedegaard, Eva Langeland

**Affiliations:** 1 Department of Welfare and Participation, Faculty of Health and Social Sciences, Western Norway University of Applied Sciences, Bergen, Norway; 2 School of Medicine, Yale University, New Haven, USA; 3 University of Bergen and Haukeland University Hospital, Bergen, Norway; 4 Department of Health and Caring Sciences, Faculty of Health and Social Sciences, Western Norway University of Applied Sciences, Bergen, Norway

**Keywords:** Individual placement and support, IPS, People with severe mental illness perspectives, Job support, Qualitative

## Abstract

**Purpose:**

To explore clients’ experiences of receiving job support from employment specialists (ESs) working with individual placement and support (IPS) in Norway. IPS is developed to help people with severe mental illness (SMI) into competitive employment as an integral component of mental health services.

**Methods:**

Using a hermeneutic phenomenological methodology, this study comprises individual semi-structured interviews with ten participants engaged in IPS at two districts psychiatric centers. Data analysis was conducted according to systematic text condensation.

**Results:**

Three themes emerged: (1) ES—a door opener? (2) Striving to sidestep a “spider web” of triggers at and away from work; and (3) Calling for a safer route.

**Conclusion:**

This study highlights the importance of ESs offering IPS clients’ opportunities to try out diverse jobs and focusing more on assessing the work environment in the jobs they place people into. Our findings imply that ESs should spend more time on building a good working alliance with both clients and employers, and pay more attention on understanding individuals’ vocational capacities and support needs at the worksite. The ES training should focus not simply on the technical processes of job development and placement, but more directly on empowering clients to stay focused on their vocational ambitions and prospects. The salutogenic model of health can help ESs to analyze whether clients experience workplaces as meaningful, manageable, and comprehensible.

## Introduction

People with severe mental illness (SMI) view employment as central to their recovery process [1–3], principally when work task is experienced as meaningful, manageable, and comprehensible [4, 5]. However, unemployment rates remain extremely high among people with SMI, especially those diagnosed with schizophrenia [6, 7]. The costs are high: significant numbers of people are at risk of loss of life’s purpose, social isolation, poverty, and even suicide [8, 9].

Over the years, efforts have been made to improve employment prospects and financial conditions for this population. More efficient vocational rehabilitation models have been developed, particularly the evidence-based individual placement and support (IPS) model, which is consistently recommended by international and national healthcare strategies [10–14] for integration into mental healthcare services, as well as “accommodations to be made at and by the workplace” ([12], p. 146). IPS is a manualized place-and-then-train approach [15], which incorporates eight core principles: (1) Focus on the goal of competitive employment, (2) zero exclusion, (3) attention to client preferences, (4) rapid job search, (5) targeted job development, (6) integration of employment services with mental health treatment, (7) personalized benefits counseling, and (8) individualized long‐term support. The principles are quantified in the validated IPS Fidelity Scale, which measures adherence to the method across different cultural contexts ([16], p. 2–3). ESs work closely with clients, clinicians, and employers, playing a pro-active role that requires good communication skills and knowledge of mental health problems, mental health services, and local workplaces within different sectors [17–20]. Over the past three decades, IPS has spread from the US to 20 countries [16, 21, 22]. In Norway, IPS programs have increasingly expanded since the early 2000s [23, 24]. In 2023, a total of 58 IPS programs were registered [25]. A recent mixed methods study, which evaluated six IPS centers in Norway, showed fair to good fidelity according to the IPS fidelity scale. Several implication challenges during the first year were identified, but the interviewed IPS clients described the ES role in positive ways [26]. Notably, results from another Norwegian study imply that more attention should be paid on the role of emotional support and involuntary hospitalization regarding the predictors of employment for people with moderate to severe mental illness [27].

Although IPS has been documented as the most efficient work integration model for people with SMI [16, 28, 29], IPS clients’ job success remains limited. Accordingly, as studies have shown a median competitive employment *rate of 55%* [30, 31], in nearly half of which cases the client loses their job or quits within six to eight months [32], the healthful employment discourse has been criticized for camouflaging a deeper understanding of the challenges IPS clients often experience in their jobs (see, e.g., [33, 34]). Nonetheless, lack of work experience and lack of use of efficient strategies toward employment are reported as the major barriers to job acquisition for people with SMI [35].

In contrast, known facilitators of employment include clarity regarding role and responsibilities [36]; job match [37–39]; caring colleagues [40]; a good working alliance between ESs and clients, which partly depends on the ESs’ competencies [19, 41–43]; training and supervision at the worksite [44]; and workplace accommodations, such as flexible scheduling/reduced hours and modified job duties/descriptions [32, 45]. The different factors may change in importance over time; for example, psychosocial factors such as job satisfaction are most crucial in the early months of employment [37, 45]. In other words, people who feel welcomed, assured, competent, and useful do better and last longer in a job, which are central health promotion features that can be understood in the light of the concept of sense of coherence [5]. More precisely, according to the salutogenic model of health, employees who experience their working conditions as consistent, balanced in terms of underload-overload, and with opportunities to participate in decision-making, will have better chances of finding their work comprehensible, manageable, and meaningful, and of improving their sense of coherence and mental health ([4], p.322), [5].

Previous IPS research has largely focused on measuring outcomes of the program, paying little attention to clients’ experiences, although some qualitative evidence of these aspects exists that indicates that IPS clients view their relationship with an ES as central to job success. Notably, studies have reported that most participants found their ES willing to step outside their professional role and provided them with “the little extra” [46], such as personal and practical support [47], and thus helped them to stay focused and move forward [48–50], and also that ESs with prior mental health experience were highly valued [51]. However, some ESs were described negatively, indicating, for instance, that they pursued unsuitable job leads [48].

Internationally, there is a knowledge gap of lived experience of receiving IPS in a job development and working phase. We need to know more about those who do not get or stay in a job. Correspondingly, in a national context more knowledge is needed about mainstream ESs’ practices from an IPS client’s perspective. Thus, the aim of this study was to explore IPS clients’ experiences of receiving job support from employment specialists (ESs) working with individual placement and support (IPS) in Norway.

## Method

This qualitative study was designed in accordance with hermeneutic phenomenological methodology, which required the researcher to be constantly open to understand the nature of lived human experience for IPS clients. In line with Husserl’s phenomenology, the researcher was attempting to capture the essence or structure of the phenomena by searching for the spoken and unspoken meanings in the participants’ various stories, as free as possible from cultural contexts and explanations [52]. Heidegger’s and Gadamer’s hermeneutic represents a dialogical method whereby the horizon of the researcher/interpreter and the phenomenon being studied are combined together [53]. In this study, both perspectives were used to open the way for re-construction of meaning related to the participants’ unique experiences receiving IPS in job development and working phases, and the creation of new knowledge about this phenomenon. Openness was critical to ensuring that the interview process to stay as close to what the participants really experienced as possible, and not to what they think they experienced. In the processes of analytical reduction of the data, the researcher was involved in de-contextualizing and re-contextualizing the material [54].

### Participants and Sampling

In the context of the implementation of IPS programs at three DPS in Bergen, Norway the data were collected in the middle of 2019. We invited a purposeful sample of IPS jobseekers/employees to participate in the study. The inclusion criteria required participants to have been engaged in a high-fidelity IPS program, for at least three months or to have dropped out from the IPS program or a paid IPS job and to be willing to talk about their IPS job support experiences. Potential participants were identified by the service providing the IPS program, represented by the leader of Hordaland’s IPS program, which used Norwegian Labour and Welfare Administration’s (NAV) register to locate the contact information (individuals’ home addresses and mobile telephone number) of potential participants (IPS clients). In total, the leader of Hordaland’s IPS program sent a short information letter about the project by mail and invited 50 individuals to contact this study’s first author by cell phone or email if they were interested in participating in the study. Due to no response the leader sent an invitation letter to 30 more individuals, and thereafter a reminder by SMS. Finally, ten individuals replied by SMS to the first author and agreed to participate, none of the one dropped out. Then the first author called the recruited participants to shortly inform about this study and her professional background as an occupational therapist/researcher, and to arrange interview appointments. Each participant received a reminder by SMS one day before the meeting. The ten participants in this study (five women and five men, with age ranging from 20 to 40 years) had been enrolled in IPS for periods lasting between eight months and three years. When the interviews were conducted, four of participants were working in part-time jobs: two were helped by their ES and one by a previous colleague, and one worked in a family business. The others were either seeking disability pension, unemployed and out of IPS, or soon to start studies.

This study was performed in line with the principles of the Declaration of Helsinki. The Regional Committee for Medical Research in Norway approved the project (#2018/1775). Standards included issues of informed consent, confidentiality, anonymity, procedure, and distribution of the results. The interviewer (first author) had no role or responsibility in the IPS program.

### Data Collection

The first author, a trained researcher and occupational therapist, conducted and audiotaped a semi-structured interview with each participant lasting approximately 1.5 to 2 h. All interviews were face to face. Two participants chose to be interviewed in an office at the DPS they were attending and eight preferred to meet in an office at the university site. The interviews started with a short conversation and introduction of the interview focus. Each participant was provided with a supplementary information letter, which included a consent form to be signed. The semi-structured interviews followed a guide, developed by the first author and one person with lived experience, which included questions such as: What is it like to be an IPS jobseeker or employee? What has been your experience of collaborating with your ES? What kind of job support have you found helpful/less helpful? Can you tell me about challenges you have experienced as an IPS jobseeker/employee? All interviews were transcribed verbatim.

### Data Analysis

The audio-recorded data were transcribed verbatim and analyzed, using Giorgi’s [55] qualitative method, as adopted in Malterud’s systematic text condensation [54]. The analysis began shortly after all interviews were completed. Common themes and meanings were identified across the individual interviews through a four-step analytic procedure [54]. In Step 1, the first and last authors (mental health nurse and researcher) read each interview transcription. To gain an overall impression of the whole, they separately listed, discussed, and agreed on the preliminary themes (e.g., a caring and encouraging ES who has time for me, challenging and long-lasting job-seeking phases, difficulties with fitting in at work). In Step 2, meaning units from the transcripts were extracted and sorted into code groups (e.g., valuing available, positive and understanding ESs, worries about how to prepare for a working life and manage social demands at work, calls for more adjustments and flexibility from employers). In Step 3, avoiding abstractions, the meaning units in each code group were condensed, and written in first person, staying close to the participants’ self-understanding, as artificial quotations expressing the differentiated meanings of the code groups. In Step 4, the first author transformed the condensates into an interpretive and analytic text presenting the most salient content related to the phenomenon grounded in the empirical data, including quotations. New headings for the code groups were developed and turned into results categories. The results were validated against the initial complete transcripts, and the interpretations were recontextualized and validated against the initial complete transcripts [54].

## Results

We found that the participants’ experiences related to the job-seeking phase involved mixed feelings about the ES role as a door opener. Furthermore, our data analysis revealed that as IPS employee the participants struggled to sidestep the “spiderweb” of triggers at and away from work. In relation to being supported into getting and keeping IPS jobs they called for “a safer route.”

### Employment Specialist – A Door Opener?

This theme addresses the participants’ various experiences of the help and encouragement they had received from their ES in relation to the processes of searching for and getting a job. Most of the participants labeled the job-seeking periods as “challenging and long.” However, they predominantly expressed contentment with what they evidently experienced as the ESs’ caring and personalized assistance in ways of exploring their job interests and helping with the job search and job interviews: a role that one participant described as being “an icebreaker.” Most participants talked about how their ES had tried to help them with expanding their comfort zone in this phase and tuned into their support needs, such as one participant with a short work history said: “The plan was to apply for jobs by myself, but as I, in that period, was a bit unbalanced, some days on the top, the next I backed out. So we agreed that she (*the ES)* should follow me” (Fred). Most participants emphasized that they appreciated their ES’s ways of communicating and bonding with them. They liked having their own ES who was easily accessible by mobile during working hours and whom they could meet at cafés, which usually meant a friendly tone in a relaxed setting. Notably, several participants found their ES to be friendly, enthusiastic, available, open, encouraging, understanding, and analytical, somewhat in contrast with the previous more negative impressions of NAV employees, whom they described as “unreachable and impersonal.” They talked about how their ES had helped them becoming more conscious of their own actions and mindset and motivated to make a change. As two of the youngest participants, said:

She usually asked me how it went: how was it going, how did I experience it. Like, okay, you’ve been to work, but how was it arriving at work and what is it like getting out the door at home—was it like you wanted to or was it like you almost had to be pushed to work? Yeah, she asked me a lot about how I was doing and how I coped with the things I’m doing, so there was an incredible focus on me. (Arne)

Yeah, I talk a bit with her *(the ES)* about what kind of help I might need in the labor market, like with perfectionism, and concerning dialogs or collaborating with others, and even how to handle conflicts, especially concerning matters outside my zone of experience, positive self-assertion, yeah, those psychosocial aspects at a workplace. (Anton)

However, embedded in some of the stories were negative experiences related to how the participants perceived their ES’s willingness, or ability, to grasp and address how the mental illness affected the participant’s work performance. As one participant said, “Like, I often felt that, no we should not talk about the illness, right, like, we should focus on work” (Jan). In the interviews there were several expressions of implicit ambivalence about the ways the ES had supported them in the job-seeking phase. For example, two participants, both with long work history, talked about how they felt their ES’s strategies, experienced as either being too offensive or too defensive, had influenced on their processes of recovery in somewhat negative ways. The following citations can illustrate these aspects:

A period I felt things were going better, but I got a down period when she (*the ES)* was pushing me. And then, as it was a bit too much, I signed out of IPS again, actually, but it wasn’t because she was too pushy, it was because I was down, then it was difficult for me seeking jobs, to be in that situation by then. (Svein)

I would prefer being in touch with her (*the ES*) more often, because then I would become more active, like, when we met only every sixth week, I fell back into my patterns like it’s no use to offer people like me a lot of time to get things done, because after a week we will slide into isolation . . . into our bubble. (Jan)

Moreover, some of the participants explicitly reported somewhat mixed and disappointing experiences of their ES ways of approaching them. They felt that their ES was disengaged, not being there for them, as one participant said:

But maybe, I’ve been not so pleased with X’s (*the ES*) personality, yeah, like the way X talks to people. X has very little time to spend on only me, like when I call, X might be busy. Also like when we have an appointment, for example, I shouldn’t be the one who is first to arrive. I had to wait for X so long, that’s something that can be very annoying, X should come at the right time, but except for that, we had a good chemistry. But for me it is very important that I have one *(ES)* whom I can always rely on. (Siv)

### Striving to Sidestep “Spiderwebs” of Triggers at and Away from Work

This theme reflects on how the participants valued the worker role and the challenges they had experienced as IPS job seekers and IPS employee. In various ways, all the participants stated that they wanted a job to structure their everyday lives with meaningful activity and to acquire an income and a respected role in the society. Mostly they regarded employment as an arena for change, offering opportunities to learn from others in an operative community, be active, stop living passively, stop thinking negatively, and build self-esteem and skills. All participants talked about how they tried to plan and train for work participation. For example, they emphasized how they strived to improve their mental health by bringing more balance into their lives and cultivating constructive routines, interests, and their social network, and by being physically active. However, a turning point in most of the participants’ stories was their worries about how to manage working life, even when enrolled in IPS, and unemployment. Various stressful and negative occurrences tended to activate their symptoms and feeling of mental distress. A mixture of internal and external triggers led to downwards spirals, in which they felt socially excluded, lonely, and bored as unemployed, and “invisible,” humiliated, disconnected, overwhelmed, while being placed or “forced” into jobs that neither matched their level of competence nor their personality and values. Some of these meanings are illustrated by the following statement:

The life I’m living now is very isolated, but, for me also to be employed would be very tricky, there will be so many triggers. A complex spiderweb of symptom-prompts, and if too many of them are activated, I will go from being a functional human being to being a nonfunctional one. There will be so many complicated facets involved, mingling, moving around in public areas. And the doings, right, I can’t stand undertaking monotonous and repetitive tasks, then things start spinning. Also, at home, as soon as I don’t manage to activate myself, the symptoms will arise. Paranoid thoughts, psychotic thoughts, very much like that. So in that sense I’m very sick I know. In my IPS period, we were very unlucky in the kinds of jobs we had contact with, which had an effect on me getting like many symptom triggers on top of each other. Of course, then things became very difficult. (Jan)

Indirectly, this quote also showed the participant’s dissatisfaction with the jobs and the support he was offered. In particular, he felt that his ES did not listen to his calls for help regarding the passive role he was given in his IPS job. He had been placed in an unfamiliar workplace that matched well with his education and skills, but as he was given work tasks below his level of competence, he felt underrated and unnoticed. He said, “I was just rambling along with younger colleagues. So, if I couldn’t be with someone in the morning, I was just sitting in the waiting room, staring at the wall.” His ES advised him to hold on to this workplace because “this was one of the few available relevant jobs” (as he recalled his ES saying), so he tried to do so, but dropped out after approximately three months. Similarly, a few participants reported feeling overlooked by their employer or their ES in respect of their outspoken calls for work adjustments related to, for instance, flexible working hours, unpredictable or unsuitable work tasks, or high social demands at work. In various ways, the participants elaborated on how their stressful IPS jobs triggered downward spirals of anxiety or psychosis, reduced their work capacity, and led to job resignation. Some aspects of these are expressed in the following two quotes:

It was very exhausting for me trying to fulfill the expectations of working eight-hour days, for me to socialize so long. I managed to do so for a period, but I then felt totally drained. It was so difficult to meet people who were irritated, not showing sympathy for my vulnerability. I had to be so on the alert about whom I might possibly meet during the workday. Because if I met someone who I felt was irritated because of me and a bit aggressive, I felt that I had to be very watchful and kind of meet them with reservation. And then I couldn’t relax. I was in a way not able to set my own boundaries. (Aslaug)

At once there were demands, at once I was expected to perform something, I became psychotic. It was very challenging, right, because you don’t want -you are expected to treat customers at the cash register, it was (just) my second day in that job, right. I had been there only one hour on my first day. And I was placed at the cash register the second day. Yeah, that’s how they do it. You are expected to be trained very rapidly, but when your voices and visual hallucinations arise, then you don’t feel on top. But I realized this was something I wanted to achieve. But I couldn’t manage to do it right then. So, that was the background for me to start trying again, even though I was so sick then. (Mari)

The participants elaborated on their attempts at coping with what they apparently experienced as work situations they were unprepared for. They strived to stretch their comfort zone; however, there were many stories about failing. For example, the two participants quoted above were out of IPS when the interviews were conducted. One of them managed to keep another challenging but stimulating IPS job for almost two years but quit after several sick leaves and was unemployed at the time of the interview. The other participant was evidently affected by her recent negative work experience in a socially demanding full-time IPS job for approximately three months; the employer did not permit part-time work, so she decided to choose a sheltered job.

### Calling for a Safer Route

Based on their experiences in the social context of the different workplaces, this theme reflects how nearly half of the participants viewed their chances of attaining and maintaining a paid job as low. This disbelief was rooted in how they experienced the role they were given as an IPS jobseeker and employee, the respective employer’s resistance of being flexible to making adjustments for their needs, and the lack of hands-on support from the ES at the work site. In the words of two of the participants:

I don’t fit into the ordinary labor market; it is too stressful for me. Working is meaningful, but I cannot manage in these circumstances. (Aslaug)

I never felt like a real employee, I never got what the other employees had. I never got a desk like the others did, I didn’t get anything of what they had, right. (Jan)

Several participants expressed distress at being placed in what they felt were unsuitable IPS jobs. Evidently, their recent negative work experience had dragged them into downward spirals. Notably, one participant, reflecting upon his previous job failures, articulated ways that he believed his ES could have helped him more, both as an IPS jobseeker and as an employee:

Less time on planning and more time on just trying out a variety of workplaces, and not taking it so seriously if this (job) didn’t work, right, maybe just try out a new one for two weeks. Like for me it would be better to test maybe like ten jobs. More flexibility, right, if one person can bear one activity, then it’s better to get people home and then out again, when there is something to do that might work out. I think there’s a lot of things with that job I had which could be fixed in very simple ways. So, I would have preferred for her (the ES) to come there and realize how it was (for me) sitting in the hallway and how ridiculous it really was. (Jan)

Regardless of their background, education, age, or experience, the participants described vocational letdowns and uncertainties in their IPS jobs. As one participant put it: “To find this: what am I doing here? Yeah, that was maybe the most challenging matter” (Mari). Similarly, another participant stated: “Yes, it has been difficult for me, wishing I could stay in a permanent job, like all the time. But for many reasons that didn’t happen. So it might be a good idea to like to get more help to stay employed” (Siv). As the above quote demonstrates, several participants reported a decline in follow-up frequency after being placed in a job, and none explicitly described on-site training. Several participants noted that they would prefer more regular contact with their ES. In Fred’s words:

I’m a person with maybe a kind of big ego, so I don’t always want to get in touch if I’m unsure of something or like, so maybe just ask if everything is going well . . . is not a big thing for me, but I like to believe that I can manage everything and not ask for help, I don’t like that, or it’s kind of uncomfortable, so it can be like even if everything seems good . . . . I’m very clever at making it look like everything is good. (Fred)

Notably, several participants also spoke about their personal development while participating in IPS. Mari had the following to say:

What I have learned the most is to trust people more. That I don’t have to do everything myself. Sometimes it’s good to have someone on your team who you can trust. At the same time I have learnt that I must in a way set my own boundaries.

## Discussion

This study revealed a variety of perceptions that can allow more insight into the helpful and unhelpful job support contributions of ESs from IPS clients’ perspectives. Firstly, in line with the literature [46–48, 51, 56] and the IPS key principles [16], the participants in this study valued having “their own” ES, whom most of them experienced as a friendly, encouraging, and accessible helper who took their preferences seriously and provided them with useful assistance in flexible ways. This is an important finding, as previous research (see, for instance, [37, 41, 42, 57, 58]) indicates that critical factors known for job success include having trusting and hopeful emotional support, the client feeling seen as a person and not as “a diagnosis,” and the ES being reachable, with consistent job focus and open-minded about the client’s vocational aspirations [26]. Additionally, in line with the previous research [41, 42, 59, 60], this study shows the importance of support away from the work site for boosting the participant’s self-confidence and ability to handle work stress. In striving to balance and fulfill workplace demands, all the participants in this study were aware of their ways of stabilizing and trying to maintain their mental health. As previous research indicates [39], the development of self-management strategies is an important aspect of a recovery journey, as is stress control at work.

Secondly, this study’s findings also illustrate important issues relating to, and the participants’ mixed feelings about, the support they received from their ES while in IPS jobs or on placements. The participants expressed, implicitly or explicitly, dissatisfaction with unmet support needs. In various ways they felt let down by their ES, colleagues, and/or employers, which is a factor known to have an impact on employee work satisfaction, work performance, and job tenure [32]. This finding echoes the debate in the IPS literature on the variable quality of ES contributions (see, for instance, [44, 59]). For example, it has been noted that higher-performing ESs tend to be more sensitive, optimistic, open-minded, person-centered, warm-hearted, affirmative, and persistent than lower-performing employment specialists, who appear to be less connected, such as by spending less time on exploring and addressing their clients’ support needs [61–65]. Notably, researchers have recommended recruiting ESs “who score high on extraversion and low on negative emotionality” (see, for instance, [20]). Moreover, previous research reveals a tendency among ESs to “cream” clients who are considered “job ready” and to feel pressured to achieve positive employment outcomes in their practice [66]. A more critical interpretation of our finding may be that some of our participants were not viewed as “employable.”

Given that most of the participants in this study had failed in their efforts to cope with the actual demands of their IPS jobs due to work task expectations that were either too high or too low, uncertainty in the worker role, too much pressure to participate socially, or overly long working days, they called for more follow-up, more emotional and practical support, and more help with workplace adjustment from their ES in terms of both quality and quantity. This is an important finding, as it shows the significance of the positive effects of a good job match and the provision of “hands on” ongoing support as needed, including contact at the worksite, with both clients and employers, as opposed to just “dropping” clients once they appear to be stable in their jobs ([36, 37], p. 154), [44].

Thirdly, this study highlights the importance of ESs offering IPS clients’ opportunities to try out diverse jobs and focusing more on assessing the work environment in the jobs they place people into. The participants calls for more follow-up in getting meaningful and manageable jobs [32]. Notably, existing qualitative research advises ESs to focus more on directing their job support toward clients struggling to fit into the work environment when planning job support. For example, according to a recent meta-ethnography [43], ESs should pay more attention to spotting the so-called “invisible work-place icebergs” (psychosocial, behavioral, and environmental workplace factors) to help IPS clients avoid “crashing” into them and, by so doing, help them keep their jobs. Likewise, to achieve a better understanding of client job support needs, the literature suggests the occupational therapy-based Person-Environment-Occupation (PEO) model [67] as a useful tool for employment specialists to analyze the dynamics between an employee’s personal capacities, the workplace environment, and an occupation’s work tasks [68].

As current research points out [60, 68], most people living with SMI are aware of their performance limitations, but find it difficult to predict the frequency, intensity, and kind of help they need in a new job. Trying out different kinds of jobs may provide an opening for IPS clients to develop their careers and grow into an identity as a worker over time [39]. Thus, individuals should be given opportunities to test their own capacities at diverse workplaces. Moreover, as noted in the literature, it is important for ES to address whether IPS clients’ self-perception change in positive ways during the vocational rehabilitation processes [32]. Significantly, it is vital that ESs adopt flexible and transparent ways of providing support, such as by documenting a client’s progress and difficulties as they move through different jobs, work environments, and life situations [60]. Moreover, the salutogenic model of health applied in this study can help ESs to analyze whether a client experiences a workplace as meaningful, manageable, and comprehensible [4]. Accordingly, this knowledge can direct ESs toward offering employers a more tailored support to assist them in enabling positive processes of work integration. More precisely, in order to have a higher sense of coherence, it is important that the employees’ working conditions are experienced as welcoming and flexible, rather than enforcing a rigid workplace environment or schedule, and offer appropriate challenges and meaningful tasks [4]. This seemed not to be the case for several participants in this study. For instance, although the participants pointed out to their ES that they had limitations when interacting socially at work, they felt they were not helped with such issues, a finding that may indicate a weaker working alliance between some ESs and participants. Moreover, several participants seemed to experience too much stress due to high job demands and a lack of job resources at the workplace and support from their ES, overloads which exacerbated their mental health issues. Also, when unemployed and without plans, the participants felt miserable, isolated, and alone, and thus experienced underload [4]. This finding shows the importance of ES’s communication and “navigating” competencies [43], so as to provide enough backing up and find jobs that offer their clients appropriate challenges: To understand clients’ social and work capacities, workplace components, and activities involved in work tasks [67]; to support them in “finding their balance” at work [36]; and by so doing, to promote individuals’ sense of coherence and mental health [4].

### Reflection on the Research Processes

Consistent with the goals and philosophy of qualitative inquiry [54], the aim of the study was not to offer results that could be widely generalized. Rather, it was to capture important insights based on these participants’ experiences and perspectives. Research questions and interpretations of participants’ narratives will always be influenced by a researcher’s horizon of understanding and experience [53]. However, as a female researcher (PhD) and occupational therapist, the first author tried to remain as open and reflexive as possible, both in the interview situation and while approaching and analyzing the data. The last author, a female researcher (PhD) and mental health nurse, read the transcripts and actively participated in the analysis and the review of the article. The second author, male and researcher (PhD) and psychologist, and the third author, male and a researcher (PhD) and psychiatrist, participated in designing the study, reviewing the article, and confirming the study results and discussion.

## Conclusion

This study highlights the importance of ESs offering IPS clients opportunities to try out diverse jobs and focusing more on assessing the work environment in the jobs they place people into. Significantly, it is vital that ESs adopt flexible and transparent ways of providing support, such as by documenting the clients’ progress and difficulties as they move through different jobs, work environments, and life situations. Our findings indicate that IPS clients’ need opportunities to test their talents and capabilities in various workplaces, suitable work challenges, and flexible workplaces that provide necessary resources to empower employees to work. The salutogenic model of health can help ESs to analyze to what extent a client experience a workplace as meaningful, manageable, and comprehensible [4].

This study implies that ESs should spend more time building a good working alliance with both clients and employers, and pay more attention on understanding individuals’ vocational capacities and support needs at the worksite. By so doing, they will be more attuned to the central IPS principles, which emphasize finding jobs that match the clients’ preferences, skills and capacities, and the workplace demands, as well as long-term support. Accordingly, to build sustainable and high-fidelity IPS programs and to ensure that the service benefits the user, the whole local organization needs to empower the ES with education and ongoing training of this evidence-based intervention. Thus, the ES training should focus not simply on the technical processes of job development and placement, but more directly on empowering clients to stay focused on their vocational ambitions and prospects.
